# *ClassifyMe*: A Field-Scouting Software for the Identification of Wildlife in Camera Trap Images

**DOI:** 10.3390/ani10010058

**Published:** 2019-12-27

**Authors:** Greg Falzon, Christopher Lawson, Ka-Wai Cheung, Karl Vernes, Guy A. Ballard, Peter J. S. Fleming, Alistair S. Glen, Heath Milne, Atalya Mather-Zardain, Paul D. Meek

**Affiliations:** 1 School of Science and Technology, University of New England, Armidale, NSW 2351, Australia; clawso21@une.edu.au (C.L.); kcheun22@une.edu.au (K.-W.C.); 2 School of Environmental and Rural Science, University of New England, Armidale, NSW 2351, Australia; kvernes@une.edu.au (K.V.); guy.ballard@dpi.nsw.gov.au (G.A.B.); peter.fleming@dpi.nsw.gov.au (P.J.S.F.); paul.meek@dpi.nsw.gov.au (P.D.M.); 3 Vertebrate Pest Research Unit, NSW Department of Primary Industries, Allingham St, Armidale, NSW 2351, Australia; heath.milne@dpi.nsw.gov.au; 4 Vertebrate Pest Research Unit, NSW Department of Primary Industries, 1447 Forest Road, Orange, NSW 2800, Australia; 5 Manaaki Whenua—Landcare Research, Private Bag 92170, Auckland 1142, New Zealand; GlenA@landcareresearch.co.nz; 6 IO Design Australia, Armidale, NSW 2350, Australia; io.atalya@gmail.com; 7 Vertebrate Pest Research Unit, NSW Department of Primary Industries, PO Box 530, Coffs Harbour, NSW 2450, Australia

**Keywords:** camera traps, camera trap data management, deep learning, ecological software, species recognition, wildlife monitoring

## Abstract

**Simple Summary:**

Camera trap wildlife surveys can generate vast amounts of imagery. A key problem in the wildlife ecology field is that vast amounts of time is spent reviewing this imagery to identify the species detected. Valuable resources are wasted, and the scale of studies is limited by this review process. The use of computer software capable of extracting false positives, automatically identifying animals detected and sorting imagery could greatly increase efficiency. Artificial intelligence has been demonstrated as an effective option for automatically identifying species from camera trap imagery. Currently available code bases are inaccessible to the majority of users; requiring high-performance computers, advanced software engineering skills and, often, high-bandwidth internet connections to access cloud services. The *ClassifyMe* software tool is designed to address this gap and provides users the opportunity to utilise state-of-the-art image recognition algorithms without the need for specialised computer programming skills. *ClassifyMe* is especially designed for field researchers, allowing users to sweep through camera trap imagery using field computers instead of office-based workstations.

**Abstract:**

We present *ClassifyMe* a software tool for the automated identification of animal species from camera trap images. *ClassifyMe* is intended to be used by ecologists both in the field and in the office. Users can download a pre-trained model specific to their location of interest and then upload the images from a camera trap to a laptop or workstation. *ClassifyMe* will identify animals and other objects (e.g., vehicles) in images, provide a report file with the most likely species detections, and automatically sort the images into sub-folders corresponding to these species categories. False Triggers (no visible object present) will also be filtered and sorted. Importantly, the *ClassifyMe* software operates on the user’s local machine (own laptop or workstation)—not via internet connection. This allows users access to state-of-the-art camera trap computer vision software in situ, rather than only in the office. The software also incurs minimal cost on the end-user as there is no need for expensive data uploads to cloud services. Furthermore, processing the images locally on the users’ end-device allows them data control and resolves privacy issues surrounding transfer and third-party access to users’ datasets.

## 1. Introduction

Passive Infrared sensor activated cameras, otherwise known as camera traps, have proved to be a tool of major interest and benefit to wildlife management practitioners and ecological researchers [1,2]. Camera traps are used for a diverse array of purposes including presence–absence studies [3,4,5], population estimates [6,7,8,9], animal behaviour studies [10,11,12], and species interactions studies [12,13,14]. A comprehensive discussion of the applications of camera trap methodologies and applications are described in sources including [15,16,17]. The capacity of camera traps to collect large amounts of visual data provides an unprecedented opportunity for remote wildlife observation; however, these same datasets incur a large cost and burden as image processing can be time consuming [2,18]. The user is often required to inspect, identify and label tens-of-thousands of images per deployment, dependent on the number of camera traps deployed. Large scale spatio-temporal studies may involve 10–100 s of cameras deployed consecutively over months to years, and the image review requirements are formidable and resource intensive. Numerous software packages have been developed over the last 20 years to help with analysing camera trap image data [19], but these methods often require some form of manual image processing. Automation in image processing has been recognised internationally as a requirement for progress in wildlife monitoring [1,2] and this has become increasingly urgent as camera trap deployment has grown over time.

The identification of information within camera trap imagery can be tackled using (a) paid staff, (b) internet crowd-sourcing, (c) citizen science, or (d) limiting the study size. All approaches involving human annotators can encounter errors due to fatigue. Using staff requires access to sufficient budget and capable personnel and constitutes an expensive use of valuable resources in terms of both time and money. The quality of species identification is likely to be high, but the time of qualified staff is otherwise lost for other tasks, such as field work and data interpretation. Internet crowd-sourcing involves out-sourcing and payment to commercial providers. This approach can be economical with fast task completion; however, there is potential for a large variation between annotators, influenced by experience and skill. Volunteer citizen scientists can also provide image annotation services typically via the access to web sites such as Zooniverse [20]. Costs are lower than employing staff, but reliable species identification might require specialized training and errors have important implications for any subsequent machine algorithms developed [21]. Limited control of data access, sharing and storage raises concerns around sensitive ecological datasets (e.g., endangered species) along with privacy legislation [22]. Nonetheless, the use of volunteers or citizen scientists has proved effective in the field of camera trapping—notably via *TEAM Network* [23] and the *Snapshot Serengeti* project [24]—but for some, taxa human identification has been shown to be problematic [25]. Meek and Zimmerman [26] discuss the challenges of using citizen science for camera trap research, particularly how managing such teams along with the data can incur enormous costs to the researchers. Limiting the design of studies by reducing the number of camera traps deployed, reviewing data for the presence of select species only, or evaluating only a proportion of the available data and archiving the remainder are unpalatable options. Such approaches constrain the data analysis methodologies available and limit the value of research findings [27,28].

To overcome the limitations of approaches outlined above, including human error and operator fatigue, we have utilised computer science to develop automated labelling. As well as being able to confirm results, key strengths of this approach, compared to existing options, include it being consistent, comparatively fast, standardised, and relatively free from biases associated with operator fatigue. Advances in computer vision have been pronounced in recent years, with successful demonstrations of image recognition in fields as diverse as autonomous cars, citrus tree detection from drone imagery, and the identification of skin cancer [29,30,31]. Recent work has also demonstrated the feasibility of Deep Learning approaches for species identification in camera trap images [32] and more widely across agricultural and ecological monitoring [33,34,35,36,37]. In the context of camera traps it is worth noting that such algorithms have been used in prototype software for this purpose since at least 2015, in projects such as *Wild Dog Alert* (https://invasives.com.au/research/wild-dog-alert/) [38]—building on earlier semi-automated species recognition algorithms [39]. The practical benefit of this research for end-users has been limited, because they cannot access software to automatically process camera trap images. We therefore developed *ClassifyMe* as a software tool to reduce the time and costs of image processing. The *ClassifyMe* software is designed to be used on constrained hardware resources—such as field laptops—although it can also be used on office workstations. This is a challenging requirement for a software application because it is required to operate across diverse computer hardware and software configurations while providing the end-user with a high-level of control and independence of their data. To elaborate on how we tackle these issues we outline the general structure and operation of *ClassifyMe* and provide an evaluation of its performance using an Australian species case study along with Supplementary Materials evaluating performance across Africa, New Zealand and North America.

## 2. Materials and Methods

### 2.1. Workflow

**Figure 1:**
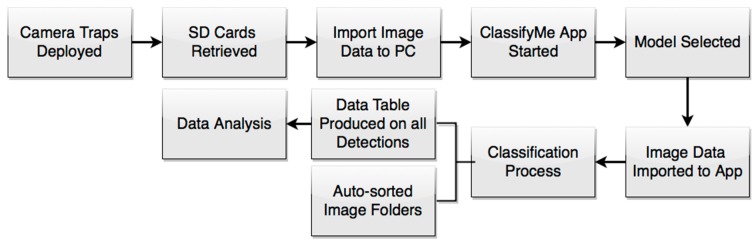
The data collection-analysis pipeline using *the ClassifyMe* software.

The software is developed so it can be installed on individual computers under an End User Licence Agreement. The intent is that the user will upload an SD card of camera trap images, select the relevant model and then run *ClassifyMe* on this dataset to automatically identify and sort the images (Figure 1).

The proposed workflow allows camera trap images to be processed on the user’s machine. This provides high level of control on the use and access to the data, alleviating concerns around the sharing, privacy and security of using web services. Furthermore, *ClassifyMe* avoids the need for the user to upload their data to cloud infrastructure, which can be prohibitive in terms of accessibility, time and cost. *ClassifyMe* adopts a ‘tethered’ service approach, whereby the user needs only intermittent internet access (every 3 months), to verify security credentials to ensure continued access to the software. The ‘tethered’ service approach was adopted as a security mechanism to obstruct misuse and the unauthorised proliferation of the software for circumstances such as poaching. A practitioner can therefore validate security credentials and download the appropriate regional identification model (e.g., New England model) prior to travel into the field. When in the field, *ClassifyMe* can be used to evaluate deployment success (e.g., after several weeks of camera trap data collection) and can be used in countries with limited or no internet connectivity. Validation services are available for approved users (e.g., ecology researchers or managers) who require extensions of the tethered renewal period.

### 2.2. Software Design Attributes

The software design and stability of *ClassifyMe* was complicated by our choice to operate solely on the user’s computer. As such, the software is capable of operating on a plethora of different operating systems and hardware designs. To limit stability issues in *ClassifyMe*, however, we have decided to only currently release and support the Windows 10^TM^ operating system, which is widely used by field ecologists. Different hardware options are supported including CPU-only and GPU; the models used by *ClassifyMe* are best supported by NDVIDIA GPU hardware and, as a result, users with this hardware will experience substantially faster processing times (up to 20 times faster per dataset).

The ‘tethered’ approach and corresponding application for software registration might be viewed as an inconvenience by some users. However, these components are essential security aspects of the software. The *ClassifyMe* software is a decentralised system; individual users access a web site, download the software and the model and then process their own data. The *ClassifyMe* web service does not see the user’s end data and—without the registration and ‘tethering’ process—the software could be copied and redistributed in an unrestricted manner. When designing *ClassifyMe*, the authors were in favour of free, unrestricted software, which could be widely redistributed. During the course of development, it occurred to the team that the software was also at risk of misuse. In particular *ClassifyMe* could be used to rapidly scan camera trap images whilst in field to detect the presence of particular species such as African elephants which are threatened by poaching [40]. To address this concern, a host of security features were incorporated into *ClassifyMe*. These features include software licencing, user validation and certification, and extensive undisclosed software security features. Disclosed security features include tethering and randomly generated licence keys, and facilities to ensure that *ClassifyMe* is used only on the registered hardware and unauthorised copying is prevented. In the event of a breach attempt, a remote shutdown of the software is initiated.

All recognition models are restricted, and approval is issued to users on a case-by-case basis. This security approach is implemented in a privacy-preserving context. The majority of security measures involve hidden internal logic along with security provisions of the communications with the corresponding *ClassifyMe* web service at https://classifymeapp.com/ (to ensure the security of communications with the end user and their data). Information provided by the user and the corresponding hardware ‘fingerprinting’ identification is performed only with user consent and all information is stored on secured encrypted databases.

A potential disadvantage of the local processing approach adopted by *ClassifyMe* is that user’s software resources are utilised, which potentially limits the scale and rate of data processing. An institutional cloud service—for instance—can auto-scale (once the data is uploaded) to accommodate data sets from hundreds of camera trap SD card simultaneously. In contrast, the *ClassifyMe* user will only be able to only process one camera trap dataset at a time. The *ClassifyMe* user will also have to implement their own data record management system—there is no database system integrated within *ClassifyMe*, which has the benefit of reducing software management complexity for end users but the disadvantage of not providing a management solution for large volumes of camera trap records. *ClassifyMe* is designed simply to review camera trap data for species identification, to auto-sort images and to export the classifications (indexed to image) to a csv file.

### 2.3. Graphical User Interface

**Figure 2:**
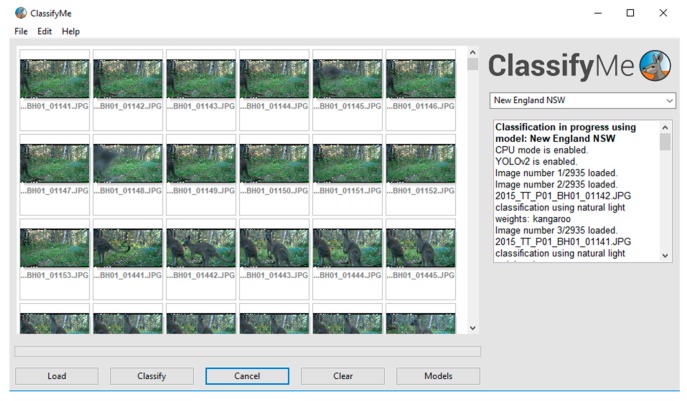
The *ClassifyMe* main user interface.

When *ClassifyMe* is initiated, the main components consist of: (a) an image banner which displays thumbnails of the camera trap image dataset, (b) a model selection box (in this example set as ‘New England NSW’), and (c) the dialogue box providing user feedback (e.g., ‘Model New England NSW loaded’)—along with a series of buttons (‘Load’, ‘Classify’, ‘Cancel’, ‘Clear’, ‘Models’) to provide the main mechanisms of user control (Figure 2).

The image banner provides a useful way for the user to visually scan the contents of the image data set to confirm that the correct data set is loaded. The ‘Models’ selection box allows users to select the most appropriate detection model for their data set. *ClassifyMe* offers facilities for multiple models to be developed and offered through the web service. A user might—for instance—operate camera trap surveys across multiple regions (e.g., New England NSW and SW USA). Selection of a specific model allows the user to adapt the model to the specific fauna of a region. Access to specific models is dependent on user approval by the *ClassifyMe* service providers. Facilities exist for developing as many classification models as required but dependent on the provision of model training datasets.

The dialogue box of *ClassifyMe* provides the primary mechanism of user interaction with the software. It provides textual responses and prompts which guide the user through use of the software and the classification process. Finally, the GUI buttons provide the main mechanism of user control. The ‘Load’ button is used to load an image dataset from the user’s files into the system; the ‘Classify’ button to start the classification of the loaded image data using the selected model; the ‘Cancel’ button to halt the current classification task, and the ‘Clear’ button to remove all current text messages from the dialogue box.

**Figure 3:**
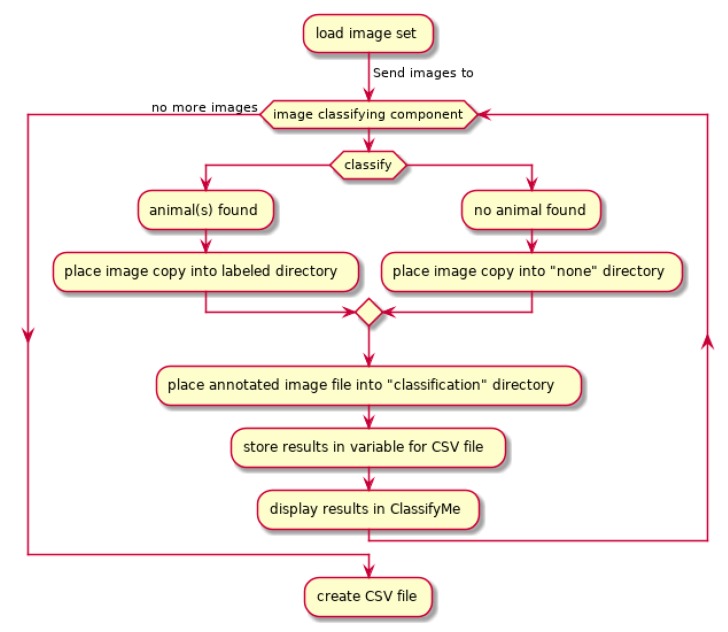
*ClassifyMe* Unified Modelling Language diagram for image classification.

When an image dataset is loaded and the classification process started (Figure 3), each image is scanned sequentially for the presence of an animal (or other category of interest) using the selected model. *ClassifyMe* automatically sorts the images into sub-directories corresponding to the most likely classification and can also automatically detect and sort images where no animal or target category is found. The results are displayed on-screen via the dialogue box which reports the classification for each image as it is processed. The full set of classification results, which includes the confidence scores for the most likely categories, is stored as a separate csv file. *ClassifyMe* creates a separate sub-directory for each new session. The full Unified Modelling Language (UML) structure of *ClassifyMe* (omitting security features) is described in Supplementary Material S1.

### 2.4. Recognition Models

The primary machine learning framework behind *ClassifyMe* is DarkNet and YOLOv2 [41]. The YOLOv2 framework is an *object detector* deep network, based on a Darknet-19 convolutional neural network structure. YOLOv2 provides access to not only a classifier (e.g., species recognition) but also a localiser (where in image) and a counter (how many animals) which facilitates multi-species detections. *ClassifyMe* at present is focused on species classification but future models could incorporate these additional capabilities due to the choice of YOLOv2. YOLOv2 is designed for high-throughput processing (40–90 frames per second) whilst achieving relatively high-accuracy (YOLOv2 544 × 544 mean Average Precision 78.6@49 frames per second on Pascal VOC 2007 dataset using a NVIDIA GeForce GTX Titan X GPU, [36]. A range of other competitive object detectors such as SSD [42], Faster R-CNN [43] and R-FCN [44] could also have been selected for this task. Framework choice was governed by a range of factors including: Accuracy of detection and classification; processing speed on general purpose hardware; model development and training requirements; ease of integration into other software packages, and licencing. Dedicated object classifiers such as ResNet [45] also provide high-accuracy performance on camera trap data [46], however such models lack the future design flexibility of an object detector.

*ClassifyMe* is designed for the end-user to install relevant models from a library accessed via the configuration panel. The model is then made available for use in the model drop-down selector box e.g., the user might install the Australian and New Zealand models via the configuration panel and when analysing a specific data set select the New Zealand model. These models are developed by the *ClassifyMe* development team. Models are developed in consultation with potential end-users and when the image data provided meets the *ClassifyMe* data requirements standard (Refer Supplementary Material S2). Importantly, *ClassifyMe* recognition models perform best when developed for the specific environment and species cohort to be encountered—and the specific camera trap imaging configuration to be used—in each study. When used outside the scope of the model, detection performance and accuracy might degrade. *ClassifyMe* is designed primarily to support end-users who have put effort into ensuring high-quality annotated datasets and who value the use of automated recognition software within their long-term study sites.

### 2.5. Model Evaluation

*ClassifyMe* has currently been developed and evaluated for five recognition models. These are Australia (New England New South Wales), New Zealand, Serengeti (Tanzania), North America (Wisconsin) and South Western USA models. The Australia (New England NSW) dataset was developed from data collected at the University of New England’s Newholme Field Laboratory, Armidale NSW. The New Zealand model was developed as part of a predator monitoring program in the context of the *Kiwi Rescue* project [47]. The Serengeti model was produced from a subset of the Snapshot Serengeti dataset [24]. The North America (Wisconsin) model was developed using the Snapshot Wisconsin dataset [48], whilst the South West USA model was developed using data provided by Caltech camera traps data collection [49]. Source datasets were sub-set according to minimum data requirements for each category (comparable to the data standard advised in Supplementary Material S2) and in light of current project developer resources.

Object detection models were developed for each dataset using YOLOv2. Hold-out test data sets were used to evaluate the performance of each model on data not used for model development. These hold-out test data sets were formulated via the random sampling of images from the project repository of images. Sample size varied based on data availability, but the preferred approach was balanced designs (equal images per class) with an 80% training-10% validation-10% testing split, with the training set used for network weight estimation, the validation set for optimizing algorithm hyper-parameters and the testing set used for obtaining model performance metrics. No further constraints were imposed, such as ensuring test data was sourced from different sites or units. This approach is reasonable for large, long-term monitoring projects involving tens to hundreds of thousands of images captured from a discrete number of cameras in fixed locations. Excessive levels of visual correlation in small, randomly sampled data subsets are generally minimal in such situations. In this case, the algorithms developed are intended to process further imagery captured from these specific cameras and locations, with model assessment approaches needing to adequately reflect this scenario. The model performance assessment does not correspond to generalised location-invariant learning; which requires a different approach, with model assessment occurring on image samples from different cameras, locations or projects. This is not the presently intended use of *ClassifyMe*, whose models are optimised to support specific large projects and not a general use case for any camera trap study. Generalised location-invariant models require further evaluation before they can be incorporated in future editions of *ClassifyMe*. Model training was performed on a Dell XPS 8930 Intel Core i7-8700 CPU @ 3.20 GHz NVIDIA GeForce GTX 1060 6 GB GPU 16 GB RAM 1.8 TB HDD drive, running a Windows 10 Professional x64 operating system using YOLOv2, via the “AlexeyAB” Windows port [50]. Training consisted of 9187 epochs, 16,000 iterations and 23 h for the natural illumination model, and 9820 epochs, 17,000 iterations and 25 h for the infrared illumination model.

## 3. Results

Overall recognition accuracies were 98.6% natural illumination, 98.7% infrared illumination for Australia (New England, NSW), 97.9% natural and infrared illumination for New Zealand, 99.0% natural and flash illumination for Serengeti, 95.9% natural illumination, 98.0% infrared illumination for North America (Wisconsin), and 96.8% natural illumination, 98.5% infrared illumination for the South West USA models. A range of model evaluation metrics were recorded including accuracy, true positive rate, positive predictive value, Matthew’s Correlation Coefficient and AUNU (Area Under the Receiver Operating Characteristic Curve of each class against the rest, using the uniform distribution) [51]. In this section, we will focus on the Australia (New England, NSW) model, further results of the other models are provided in Supplementary Material S3.

The Australian (New England, NSW) consisted of nine recognition classes and a total of 8900 daylight illumination images and 8900 infrared illumination images. Specific details of the Australian (New England, NSW) data set are provided in Table 1. Observe that the models developed only distinguish between visually distinct classes, the current versions of *ClassifyMe* models do not perform fine-grained recognition between visually similar classes, such as different species of Macropods. The component-based software design of *ClassifyMe* allows the incorporation of such fine-grained recognition models if they are developed in the future. Another important consideration is that model evaluation has been performed for ‘in-bag’ samples, that is, the data was sourced from particular projects with large annotated data sets and the model developed is intended for use only within this project and network of cameras to automate image review. The application of the models to ‘out-of-bag’ samples from other sites or projects is not intended and can produce unstable recognition accuracy.

**Figure 4:**
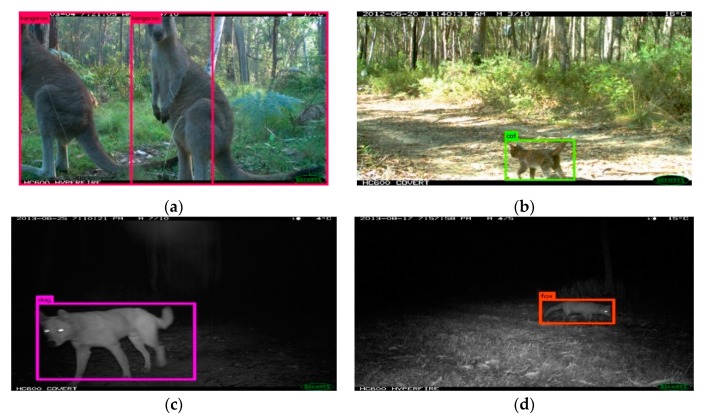
Detection Image examples from the New England dataset. (**a**) Macropod (Kangaroo), (**b**) Cat, (**c**) Dingo (dog) and (**d**) Fox.

As previously stated, model performance was assessed using a randomly held-out test data set; the detection summary (Table 2), the confusion matrix of the specific category performance (Table 3), and the model performance metrics were evaluated (Table 4) using PyCM [52]. Figure 4 displays examples of detection outputs, including the rectangle detection box that is overlaid on the location of the animal in the image and the detected category.

The results of our testing indicate that *ClassifyMe* provides a high level of performance which is accessible across a wide range of end-user hardware with minimal configuration requirements.

## 4. Discussion

### 4.1. Key Features and Benefits

*ClassifyMe* is the first application of its kind, it provides a software tool which allows field ecologists and wildlife managers access to the latest advances in artificial intelligence. Practitioners can utilise *ClassifyMe* to automatically identify, filter and sort camera trap image collections according to categories of interest. Such a tool fills a major gap in the operational requirements of all camera trap users irrespective of their deployments.

There are additional major benefits to localised processing on the end-user’s device. Most importantly, the local processing offered by *ClassifyMe* provides a high degree of privacy protection of end-user data. By design, *ClassifyMe* does not transfer classification information of user image data back to third parties, rather, all the processing of the object recognition module is performed locally, with minimal user information transferred back, via encryption, to the web service. The information transferred to the web service concerns the initial registration and installation process and the on-going verification services aimed at disrupting un-authorised distribution (which is targeted specifically at poachers and similar mis-uses of *ClassifyMe* software). These privacy and data control features are known to be appealing to many in our wider network of ecological practitioners, because transmitting and sharing images with third parties compromises (1) human privacy when images contain people, (2) the location of sensitive field equipment, and (3) the location of rare and endangered species that might be targeted by illegal traffickers. Researchers and wildlife management groups also often want control over the end-use of their data and sometimes have concerns about the unforeseen consequences of unrestricted data sharing.

### 4.2. Software Comparisons

At present, there are few alternatives to *ClassifyMe* for the wildlife manager wanting to implement artificial intelligence technologies for the automated revision of their camera trap images. The most relevant alternative is the MLWIC: Machine Learning for Wildlife Image Classification in R package [53]. The MLWIC package provides the option to run pre-trained models, and also for the user to develop their own recognition models suited to their own data sets. Whilst of benefit to a subset of research ecologists skilled with R, the approach proposed by Tabak et al. [53] is not accessible to a wider audience as it requires a considerable investment of time and effort in mastering the intricacies of the R Development Language and Environment, along with the additional challenges of hardware and software configuration associated with this software. Integration of the MLWIC package within R is sensible if the user wants to incorporate automated image classification within their own workflows. However, such automated image recognition services are already offered in other leading machine learning frameworks, particularly TensorFlow [54] and PyTorch [55]. Such frameworks offer extensive capabilities with much more memory efficient processing for a similar investment in software programming know-how (Python) and hardware configuration. In fact, our wider research team routinely uses TensorFlow and PyTorch—along with other frameworks such as DarkNet19 [41]—for camera-trap focused research. Integration with R is straight-forward, via exposure to a web-service API or via direct export of framework results as csv files. Within R, there are Python binding libraries which also allow access to Python code from within R and the TensorFlow interface package [56] also provides a comparatively easy way of accessing the full TensorFlow framework from within R. In summary, there a range of alternative options to the MLWIC package which are accessible with programming knowledge. AnimalFinder [19] is a MATLAB 2016a script available to assist with the detection of animals in time-lapse sequence camera trap images. This process is—however—semi-automated, and does not provide species identification, it also requires access to a MATLAB software licence and corresponding software scripting skills. AnimalScanner [57] is a similar software application providing both a MATLAB GUI and a command line executable to scan sequences of camera trap images and identify three categories (empty frames, humans or animals), based on foreground object segmentation algorithms coupled with deep learning.

The Wildlife Insights (https://wildlifeinsights.org) [58] promises to provide cloud-based analysis services, including automated species recognition. The eMammal project provides both a cloud service and the Leopold desktop application [59]. The Leopold eMammal desktop application uses computer vision technology to search for cryptic animals within a sequence and places a bounding box around the suspected animal [60]. The objectives and functions of eMammal are—however—quite broad, and support citizen science identifications, expert review, data curation and training within the context of monitoring programs and projects. This approach is very different from the approach adopted by *ClassifyMe*, which is a dedicated, on-demand application focused on automated species recognition on a user’s local machine with no requirement to upload datasets to third-party sources. The iNaturalist project (https://www.inaturalist.org) [61] is of a similar nature to eMammal but focused on digital or smartphone camera-acquired imagery from contributors across the world, and uses deep learning convolutional neural network models to perform image recognition within its cloud platform to assist with review by citizen scientists. Whilst very useful with a wide user base, iNaturalist does not specifically address the domain challenges of camera trap imagery. Motion Meerkat is a software application which also utilises computer vision in the form of mixture of Gaussian models to detect motion in videos which reduces the number of hours required for researcher review [62]. DeepMeerkat provides similar functionality using convolutional neural networks to monitor for the presence of specific objects (e.g., hummingbirds) in videos [63]. There is a further, wide range of software available including Renamer [64] and VIXEN [65] to support camera trap data management. Young, Rode-Margono and Amin [66] have provided a detailed review of currently available camera trap software options.

### 4.3. Model Development

An important design decision of *ClassifyMe* was to not allow end-users to train their own models. This is in contrast to software such as the MLWIC package. The decision was motivated by both legal aspects and quality control as opposed to commercial reasons. Of particular concern is use of the software to determine field locations of prized species that poachers could then target. These concerns are valid, with recent calls having been made for scientists to restrict publishing location data of highly sought-after species in peer-reviewed journals [67]. Such capabilities could be of use to technologically inclined poachers, and providing such software—along with the ability to modify that software—presented a number of potential legal issues. Similar concerns exist concerning human privacy legislation [22,68]. The strict registrations, legal and technological controls implemented within *ClassifyMe* are designed to minimise risk of misuse.

Allowing end-users to train their own models also presents quality control issues. The deep networks utilised within *ClassifyMe* (and similar software) are difficult to train to optimal performance and reliability. Specialised hardware and its configuration are also required for deep learning frameworks, which can be challenging even for computer scientists. Data access and the associated labelling of datasets is another major consideration; many users might not have sufficient sighting records nor the resources to label their datasets. The risk of developing and deploying a model which provides misleading results in practice is high—with quite serious potential consequences for wildlife observation programs. Schneider, Taylor and Kremer [69] compared the performance of the YOLOv2 and Faster R-CNN object detectors on camera trap imagery. The YOLOv2 detector performed quite poorly with an average accuracy of 43.3% ± 14.5% (compared to Faster R-CNN which had an accuracy of 76.7% ± 8.31%) on the Gold-Standard Snapshot Serengeti dataset. The authors suggested that the low performance was due to limited data. Our results clearly indicate that YOLOv2 can perform well with strict data quality control protocols. Furthermore, the *ClassifyMe* YOLOv2 model is most effective at longer-term study sites, where the model has been calibrated using annotated data specific to the study site. *ClassifyMe* is also designed to integrate well with a range of other object detection frameworks including Faster-RCNN which is utilised within the software development team for research purposes. Future editions *of ClassifyMe* might also explore the use of other detection frameworks or customised algorithms based on our on-going research focused on ‘out-of-bag’ models, suited for general use as well as the fine-grained recognition of similar species.

*ClassifyMe* resolves the issue of model development for practitioners by out-sourcing model development to domain experts who specialise in the development of such technology in collaborative academic and government joint research programs. Users can request model development, either for private use via a commercial contract, or for public use—which is free—and on the provision of image data sets to a protocol standard, the model will be developed and assessed for deployment as a *ClassifyMe* model library. *ClassifyMe* is designed to enable the selection of a suitably complex model to ensure good classification performance, but to also enable storage, computation and processing within a reasonable time frame (benchmark range 1–1.5 s per image, Intel i7 16 GB RAM) on end user computers. Cloud-based solutions, such as those used in the *Kiwi Rescue* and *Wild Dog Alert* programs, have the capacity to store data in a central location using a larger neural network structure on high-performance computer infrastructure. Such infrastructure is costly to run and is not ideal for all end-users.

## 5. Conclusions

Camera trapping is commonly used to survey wildlife throughout the world, but its Achilles-heel is the huge time and financial costs of processing data, together with the risk of human error during processing tasks. The integration of computer science and computer vision in camera trap image analysis has led to considerable advances for camera trap practitioners. The development of automated image analysis systems has filled an important gap between capturing image data in the field and analysing that data so it can be used in management decision making. *ClassifyMe* is a tool of un-matched capability, specifically for field-based camera trap practitioners and organisations across the world.

## Figures and Tables

**Table 1:** Composition of New England, New South Wales, Australia data set. Data was partitioned according to ‘Natural’ daylight illumination and ‘IR’ Infrared Illumination along with Category.

Category	Natural Sample Size (Training) {Validation} [Test]	Infrared Sample Size (Training) {Validation} [Test]
Cat	(800) {100} [100]	(800) {100} [100]
Dog	(800) {100} [100]	(800) {100} [100]
Fox	(800) {100} [100]	(800) {100} [100]
Human	(800) {100} [100]	(800) {100} [100]
Macropod	(800) {100} [100]	(800) {100} [100]
Sheep	(800) {100} [100]	(800) {100} [100]
Vehicle	(800) {100} [100]	(800) {100} [100]
Other	(800) {100} [100]	(800) {100} [100]
NIL	(800) {0} [100]	(800) {0} [100]

**Table 2:** Detection Summary results: New England NSW model (daylight). Randomly selected model training dataset with 800 images per class. Using threshold (Th = 0.24) to achieve a mean average precision (mAP) = 0.896067 (89.61%), 2967 detections, 993 unique truth count, and average Intersection of Union (IoU) = 75.04% and 902 True positives, 69 False Positives and 91 False Negatives. Total detection time was 20 s.

Class	Average Precision
Cat	99.65%
Dog	90.91%
Fox	90.91%
Human	90.91%
Macropod	80.87%
Sheep	86.46%
Vehicle	100.00%
Other	77.14%

**Table 3:** Confusion Matrix: New England NSW (natural illumination) model as assessed on a randomly selected hold-out test dataset.

**Predicted**	**Actual**										
		**Cat**	**Dog**	**Fox**	**Human**	**Macropod**	**NIL**	**Other**	**Sheep**	**Vehicle**	***Precision***
	Cat	100	0	0	0	0	0	0	0	0	1.00
	Dog	0	100	0	0	0	0	0	0	0	1.00
	Fox	0	0	99	0	0	0	3	0	0	0.97
	Human	0	0	0	100	0	0	0	0	0	1.00
	Macropod	0	0	1	0	97	0	1	0	0	0.98
	NIL	0	0	0	0	2	100	8	0	0	0.91
	Other	0	0	0	0	0	0	91	0	0	1.00
	Sheep	0	0	0	0	1	0	0	100	0	0.99
	Vehicle	0	0	0	0	0	0	0	0	100	1.00
	*Recall*	1.00	1.00	0.99	1.00	0.97	1.00	0.91	1.00	1.00	Overall Model Accuracy: 0.99

**Table 4:** Key Test Metrics of the New England, NSW (natural illumination) test data set. Note: AUNU denotes Area Under Receiver Operating Characteristic Curve comparing each class against rest using a uniform distribution.

Metric	Magnitude
Overall Accuracy	0.98556
Overall Accuracy Standard Error	0.00398
95% Confidence Interval	[0.97776,0.99335]
Error Rate	0.01444
Matthews Correlation Coefficient	0.98388
True Positive Rate (Macro)	0.98556
True Positive Rate (Micro)	0.98556
Positive Predictive Value (Macro)	0.98655
Positive Predictive Value (Micro)	0.98556
AUNP	0.99187

## Supplementary Materials

The following are available online at https://www.mdpi.com/2076-2615/10/1/58/s1, Supplementary Material S1: *ClassifyMe* UML Structure Diagram, Supplementary Material S2: Data Presentation Standard for *ClassifyMe* Software, Supplementary Material S3: *ClassifyMe* Model Assessments, Supplementary Material S4: NewEnglandDay.cfg YOLOv2 configuration file.

## References

[1] Meek P.D., Fleming P., Ballard A.G., Banks P.B., Claridge A.W., McMahon S., Sanderson J., Swann D.E., Meek P.D., Ballard G.A., Banks P.B., Claridge A.W., Fleming P.J.S., Sanderson J.G., Swann D. (2014). Putting contemporary camera trapping in focus. Camera Trapping in Wildlife Research and Management.

[2] Meek P.D., Ballard G.A., Banks P.B., Claridge A.W., Fleming P.J.S., Sanderson J.G., Swann D. (2015). Camera Trapping in Wildlife Research and Monitoring.

[3] Khorozyan I.G., Malkhasyan A.G., Abramov A.G. (2008). Presence–absence surveys of prey and their use in predicting leopard (*Panthera pardus*) densities: A case study from Armenia. Integr. Zool..

[4] Gormley A.M., Forsyth D.M., Griffioen P., Lindeman M., Ramsey D.S., Scroggie M.P., Woodford L. (2011). Using presence-only and presence-absence data to estimate the current and potential distributions of established invasive species. J. Appl. Ecol..

[5] Ramsey D.S.L., Caley P.A., Robley A. (2015). Estimating population density from presence-absence data using a spatially explicit model. J. Wildl. Manag..

[6] Karanth K.U. (1995). Estimating tiger *Panthera tigris* populations from camera-trap data using capture—Recapture models. Biol. Conserv..

[7] Trolle M., Kéry M. (2003). Estimation of ocelot density in the Pantanal using capture-recapture analysis of camera-trapping data. J. Mammal..

[8] Jackson R.M., Roe J.D., Wangchuk R., Hunter D.O. (2006). Estimating snow leopard population abundance using photography and capture-recapture techniques. Wildl. Soc. Bull..

[9] Gowen C., Vernes K., Meek P.D., Ballard G.A., Banks P.B., Claridge A.W., Fleming P.J.S., Sanderson J.G., Swann D. (2014). Population estimates of an endangered rock wallaby, *Petrogale penicillata*, using time-lapse photography. Camera Trapping: Wildlife Management and Research.

[10] Vernes K., Smith M., Jarman P., Meek P.D., Ballard G.A., Banks P.B., Claridge A.W., Fleming P.J.S., Sanderson J.G., Swann D. (2014). A novel camera-based approach to understanding the foraging behaviour of mycophagous mammals. Camera Trapping in Wildlife Research and Management.

[11] Vernes K., Jarman P. (2014). Long-nosed potoroo (*Potorous tridactylus*) behaviour and handling times when foraging for buried truffles. Aust. Mammal..

[12] Vernes K., Sangay T., Rajaratnam R., Singye R. (2015). Social interaction and co- occurrence of colour morphs of the Asiatic golden cat, Bhutan. Cat News.

[13] Meek P.D., Zewe F., Falzon G. (2012). Temporal activity patterns of the swamp rat (*Rattus lutreolus*) and other rodents in north-eastern New South Wales, Australia. Aust. Mammal..

[14] Harmsen B.J., Foster R.J., Silver S.C., Ostro L.E.T., Doncaster C.P. (2009). Spatial and temporal interactions of sympatric jaguars (*Panthera onca*) and pumas (*Puma concolor*) in a neotropical forest. J. Mammal..

[15] Linkie M., Ridout M.S. (2011). Assessing tiger–prey interactions in Sumatran rainforests. J. Zool..

[16] O’Connell A.F., Nichols J.D., Karanth K.U. (2011). Camera Traps in Animal Ecology Methods and Analyses.

[17] Meek P.D., Ballard G., Claridge A., Kays R., Moseby K., O’Brien T., O’Connell A., Sanderson J., Swann D.E., Tobler M. (2014). Recommended guiding principles for reporting on camera trapping research. Biodivers. Conserv..

[18] Rovero F., Zimmermann F. (2016). Camera Trapping for Wildlife Research.

[19] Price Tack J.L.P., West B.S., McGowan C.P., Ditchkoff S.S., Reeves S.J., Keever A.C., Grand J.B. (2016). AnimalFinder: A semi-automated system for animal detection in time-lapse camera trap images. Ecol. Inform..

[20] Zooniverse. https://zooniverse.org.

[21] Zhang J., Wu X., Sheng V.S. (2016). Learning from crowdsourced labeled data: A survey. Artif. Intell. Rev..

[22] Meek P.D., Butler D., Meek P.D., Ballard G.A., Banks P.B., Claridge A.W., Fleming P.J.S., Sanderson J.G., Swann D. (2014). Now we can “see the forest and the trees too” but there are risks: Camera trapping and privacy law in Australia. Camera Trapping in Wildlife Research and Management.

[23] Ahumada J.A., Silva C.E., Gajapersad K., Hallam C., Hurtado J., Martin E., McWilliam A., Mugerwa B., O’Brien T., Rovero F. (2011). Community structure and diversity of tropical forest mammals: Data from a global camera trap network. Philos. Trans. R. Soc. Lond. B Biol. Sci..

[24] Swanson A., Kosmala M., Lintott C., Simpson R., Smith A., Packer C. (2015). Snapshot Serengeti, high-frequency annotated camera trap images of 40 mammalian species in an African savanna. Sci. Data.

[25] Meek P.D., Vernes K., Falzon G. (2013). On the reliability of expert identification of small-medium sized mammals from camera trap photos. Wildl. Biol. Pract..

[26] Meek P.D., Zimmerman F., Rovero F.A.Z.F. (2016). Camera traps and public engagement. Camera Trapping for Wildlife Research.

[27] Claridge A.W., Paull D.J., Meek P.D., Ballard G.A., Banks P.B., Claridge A.W., Fleming P.J.S., Sanderson J.G., Swann D.E. (2014). How long is a piece of string? Camera trapping methodology is question dependent. Camera Trapping Wildlife Management and Research.

[28] Swann D.E., Perkins N., Meek P.D., Ballard G.A., Banks P.B., Claridge A.W., Fleming P.J.S., Sanderson J.G., Swann D. (2014). Camera trapping for animal monitoring and management: A review of applications. Camera Trapping in Wildlife Research and Management.

[29] Zhang X., Yang W., Tang X., Liu J. (2018). A fast learning method for accurate and robust lane detection using two-stage feature extraction with YOLO v3. Sensors.

[30] Csillik O., Cherbini J., Johnson R., Lyons A., Kelly M. (2018). Identification of Citrus Trees from Unmanned Aerial Vehicle Imagery Using Convolutional Neural Networks. Drones.

[31] Esteva A., Kuprel B., Novoa R.A., Ko J., Swetter S.M., Blau H.M., Thrun S. (2017). Dermatologist-level classification of skin cancer with deep neural networks. Nature.

[32] Norouzzadeh M.S., Nguyen A., Kosmala M., Swanson A., Palmer M.S., Packer C., Clune J. (2018). Automatically identifying, counting, and describing wild animals in camera-trap images with deep learning. Proc. Natl. Acad. Sci. USA.

[33] Ferentinos K.P. (2018). Deep learning models for plant disease detection and diagnosis. Comput. Electron. Agric..

[34] Qin H., Li X., Liang J., Peng Y., Zhang C. (2016). DeepFish: Accurate underwater live fish recognition with a deep architecture. Neurocomputing.

[35] Chabot D., Francis C.M. (2016). Computer-automated bird detection and counts in high-resolution aerial images: A review. J. Field Ornithol..

[36] Valan M., Makonyi K., Maki A., Vondráček D., Ronquist F. (2019). Automated taxonomic identification of insects with expert-level accuracy using effective feature transfer from convolutional networks. Syst. Biol..

[37] Xue Y., Wang T., Skidmore A.K. (2017). Automatic counting of large mammals from very high resolution panchromatic satellite imagery. Remote Sens..

[38] Meek P.D., Ballard G.A., Falzon G., Williamson J., Milne H., Farrell R., Stover J., Mather-Zardain A.T., Bishop J., Cheung E.K.-W. (2019). Camera Trapping Technology and Advances: Into the New Millennium. Aust. Zool..

[39] Falzon G., Meek P.D., Vernes K., Meek P.D., Ballard G.A., Banks P.B., Claridge A.W., Fleming P.J.S., Sanderson J.G., Swann D. (2014). Computer Assisted Identification of Small Australian Mammals in Camera Trap Imagery.

[40] Bennett E.L. (2015). Legal ivory trade in a corrupt world and its impact on African elephant populations. Conserv. Biol..

[41] Redmon J., Farhadi A. YOLO9000: Better, Faster, Stronger. Proceedings of the IEEE Conference on Computer Vision and Pattern Recognition.

[42] Liu W., Anguelov D., Erhan D., Szegedy C., Reed S., Fu C.Y., Berg A.C. (2016). SSD: Single Shot Multibox Detector.

[43] Ren S., He K., Girshick R., Sun J. (2017). Faster R-CNN: Towards real-time object detection with region proposal networks. IEEE Trans. Pattern Anal. Mach. Intell..

[44] Dai J., Li Y., He K., Sun J. (2016). R-fcn: Object detection via region-based fully convolutional networks. Adv. Neural Inf. Process. Syst..

[45] He K., Zhang X., Ren S., Sun J. Deep residual learning for image recognition. Proceedings of the IEEE Conference on Computer Vision and Pattern Recognition.

[46] Gomez Villa A., Salazar A., Vargas F. (2017). Towards automatic wild animal monitoring: Identification of animal species in camera-trap images using very deep convolutional neural networks. Ecol. Inform..

[47] Falzon G., Glen A. Developing image recognition software for New Zealand animals. Proceedings of the 31st Australasian Wildlife Management Society Conference.

[48] Willi M., Pitman R.T., Cardoso A.W., Locke C., Swanson A., Boyer A., Veldthuis M., Fortson L. (2019). Identifying animal species in camera trap images using deep learning and citizen science. Methods Ecol. Evol..

[49] Beery S., Van Horn G., Perona P. Recognition in Terra Incognita. Proceedings of the European Conference on Computer Vision (ECCV).

[50] “AlexeyAB” Darknet Windows Port. https://github.com/AlexeyAB/darknet.

[51] Ferri C., Hernández-Orallo J., Modroiu R. (2009). An experimental comparison of performance measures for classification. Pattern Recognit. Lett..

[52] Haghighi S., Jasemi M., Hessabi S., Zolanvari A. (2018). PyCM: Multiclass confusion matrix in Python. J. Open Source Softw..

[53] Tabak M.A., Norouzzadeh M.S., Wolfson D.W., Sweeney S.J., Vercauteren K.C., Snow N.P., Halseth J.M., Di Salvo P.A., Lewis J.S., White M.D. (2019). Machine learning to classify animal species in camera trap images: Applications in ecology. Methods Ecol. Evol..

[54] Abadi M., Barham P., Chen J., Chen Z., Davis A., Dean J., Devin M., Ghemawat S., Irving G., Isard M. (2016). TensorFlow: A system for large-scale machine learning. OSDI.

[55] Paszke A., Gross S., Chintala S., Chanan G., Yang E., DeVito Z., Desmaison A., Antiga L., Lerer A. Automatic differentiation in PyTorch. Proceedings of the 31st Conference on Neural Information Processing Systems.

[56] Allaire J.J.T.Y. (2017). TensorFlow: R Interface to TensorFlow. https://cran.r-project.org/package=tensorflow.

[57] Yousif H., Yuan J., Kays R., He Z. (2019). Animal Scanner: Software for classifying humans, animals, and empty frames in camera trap images. Ecol. Evol..

[58] Ahumada J.A., Fegraus E., Birch T., Flores N., Kays R., O’Brein T.G., Palmer J., Schuttler S., Zhao J.Y., Jetz W. (2019). Wildlife insights: A platform to maximize the potential of camera trap and other passive sensor wildlife data for the planet. Environ. Conserv..

[59] Forrester T., McShea W.J., Keys R.W., Costello R., Baker M., Parsons A. eMammal–citizen science camera trapping as a solution for broad-scale, long-term monitoring of wildlife populations. Proceedings of the 98th Annual Meeting Ecological Society of America Sustainable Pathways: Learning from the Past and Shaping the Future.

[60] He Z., Kays R., Zhang Z., Ning G., Huang C., Han T.X., Millspaugh J., Forrester T., McShea W. (2016). Visual informatics tools for supporting large-scale collaborative wildlife monitoring with citizen scientists. IEEE Circuits Syst. Mag..

[61] iNaturalist. https://www.inaturalist.org.

[62] Weinstein B.G. (2015). MotionMeerkat: Integrating motion video detection and ecological monitoring. Methods Ecol. Evol..

[63] Weinstein B.G. (2018). Scene-specific convolutional neural networks for video-based biodiversity detection. Methods Ecol. Evol..

[64] Harris G., Thompson R., Childs J.L., Sanderson J.G. (2010). Automatic storage and analysis of camera trap data. Bull. Ecol. Soc. Am..

[65] Ramachandran P., Devarajan K. (2018). ViXeN: An open-source package for managing multimedia data. Methods Ecol. Evol..

[66] Young S., Rode-Margono J., Amin R. (2018). Software to facilitate and streamline camera trap data management: A review. Ecol. Evol..

[67] Lindenmayer D., Scheele B. (2017). Do not publish. Science.

[68] Butler D., Meek P.D. (2013). Camera trapping and invasions of privacy: An Australian legal perspective. Torts Law J..

[69] Schneider S., Taylor G.W., Kremer S. Deep Learning Object Detection Methods for Ecological Camera Trap Data. Proceedings of the 2018 15th Conference on Computer and Robot Vision (CRV).

