# Supplementary material for: ClassifyMe: A Field-Scouting Software for the Identification of Wildlife in Camera Trap Images
Source: Animals (Basel). 2019 Dec 27;10(1):58. doi: 10.3390/ani10010058 (PMC7022311; doi:10.3390/ani10010058)
Supplement: Supplementary file 1 [file animals-10-00058-s001.zip › sup/ClassifyMe_SupplementaryMaterial S1.docx]

**Supplementary Material S1: ClassifyMe UML Structure Diagram**

**
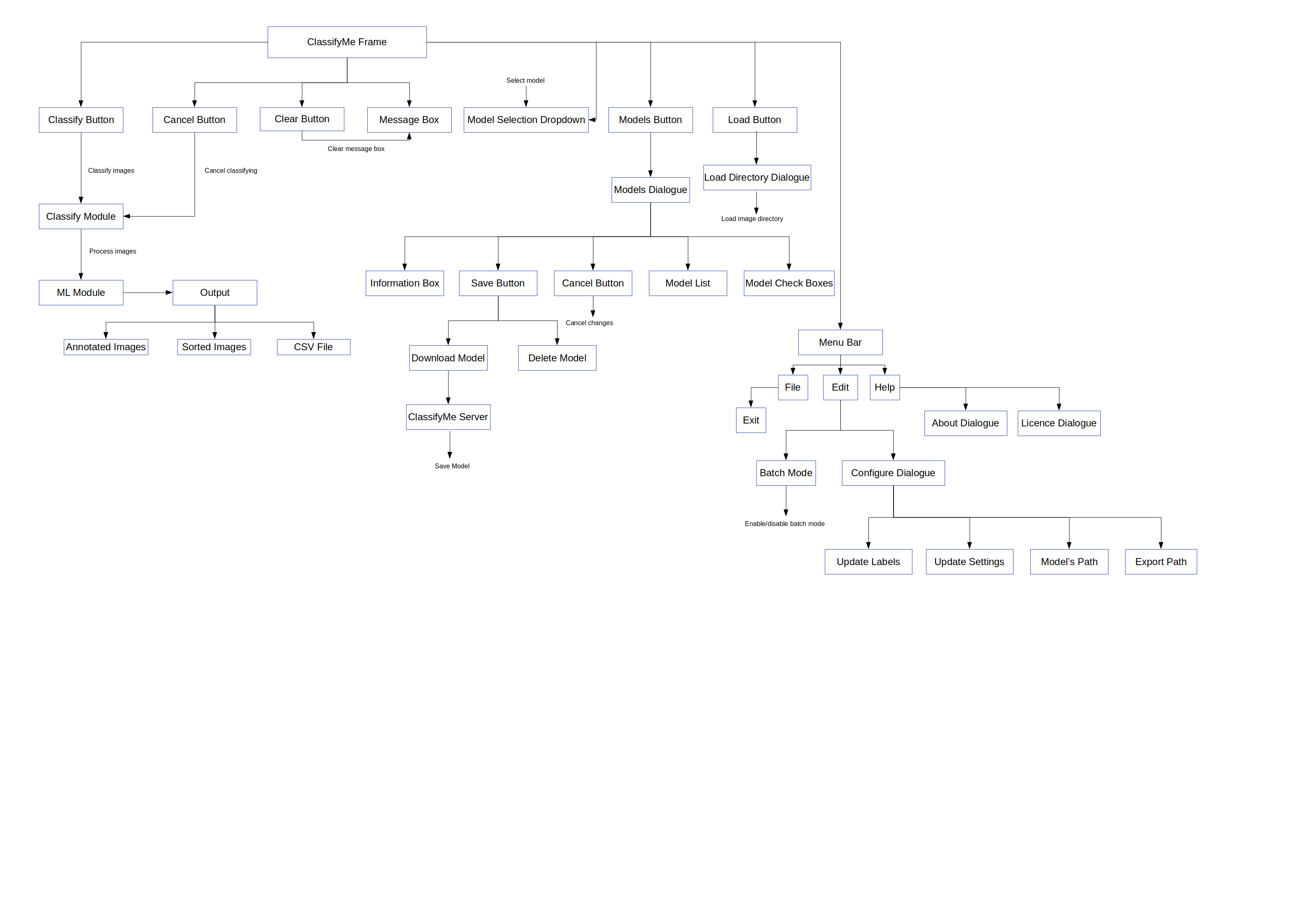
**

**Figure S1.1: ClassifyMe Block diagram**

**
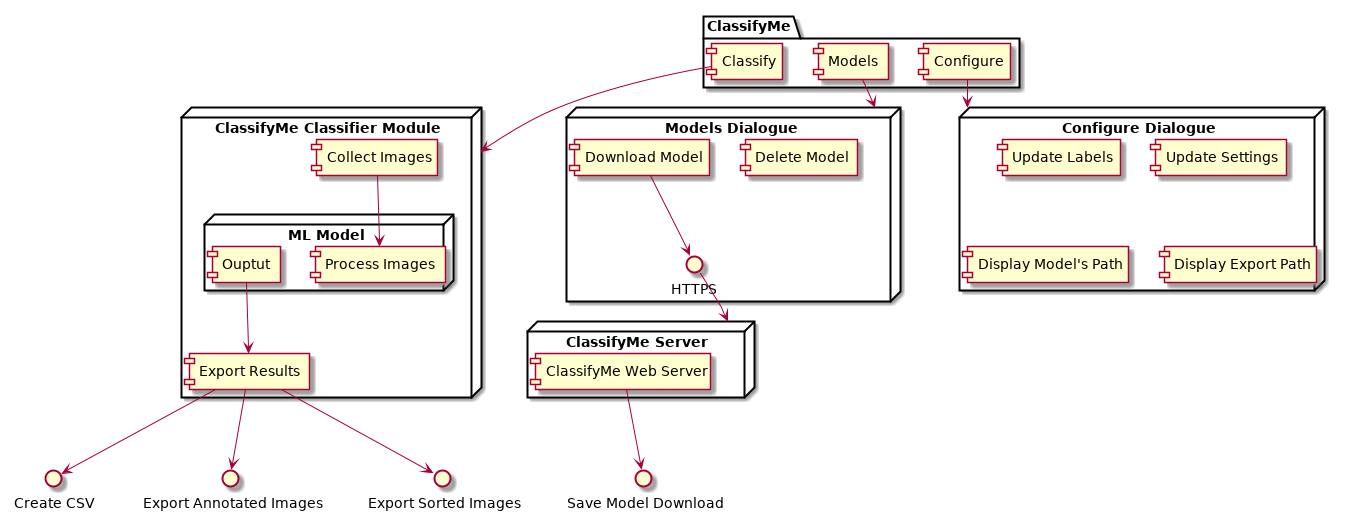
**

**Figure S1.2: ClassifyMe Basic UML (Security Removed)**


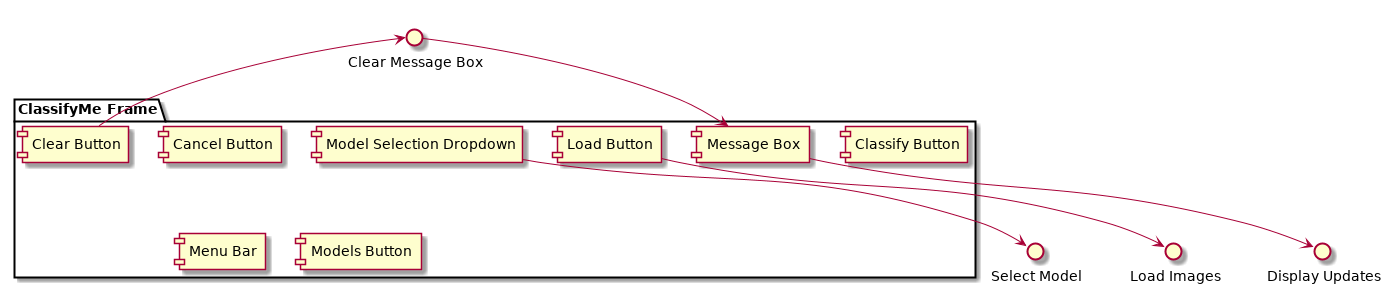


**Figure S1.3: ClassifyMe Frame/GUI UML**


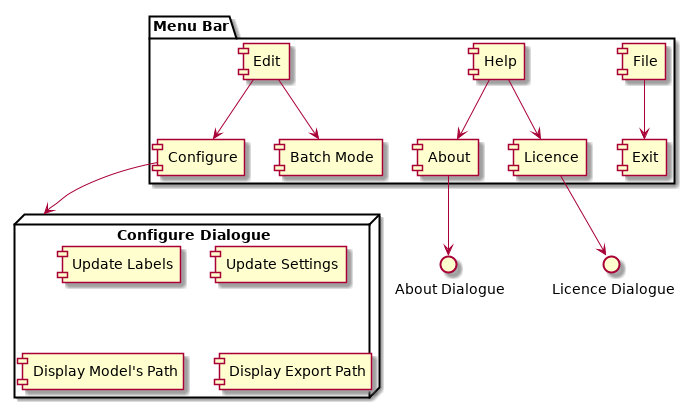


**Figure S1.4: ClassifyMe Menu Bar & Configuration Dialogue**


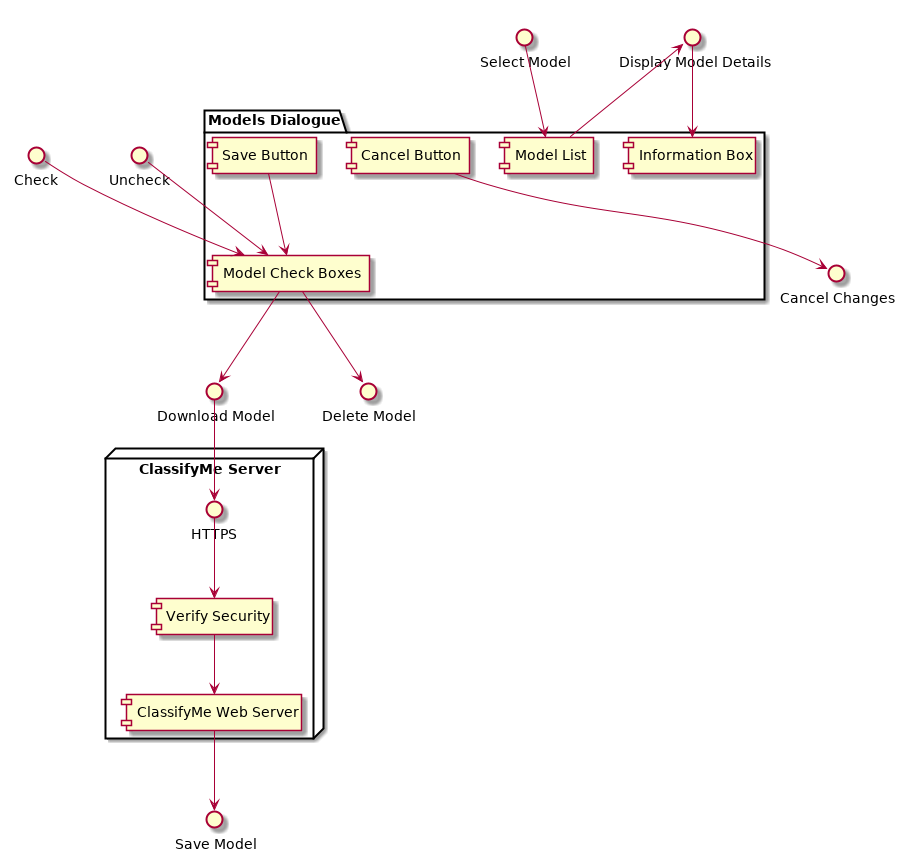


**Figure S1.5: ClassifyMe Model Management.**


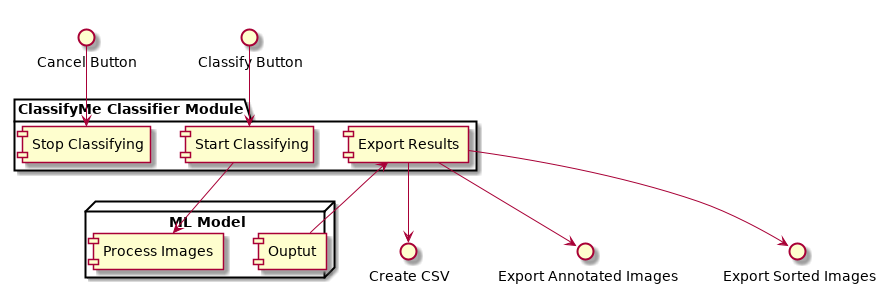


**Figure S1.6: ClassifyMe Image Classification.**
