# Supplementary material for: ClassifyMe: A Field-Scouting Software for the Identification of Wildlife in Camera Trap Images
Source: Animals (Basel). 2019 Dec 27;10(1):58. doi: 10.3390/ani10010058 (PMC7022311; doi:10.3390/ani10010058)
Supplement: Supplementary file 1 [file animals-10-00058-s001.zip › sup/ClassifyMe_SupplementaryMaterial S3.docx]

**Supplementary Material S3: *ClassifyMe* Model Assessments**

This appendix reports on the results of *ClassifyMe* applied in 5 different environments. These environments are: (i) New England Tablelands, New South Wales, Australia; (ii) Wisconsin, USA; (iii) South Western USA; (iv) Serengeti Plains, Tanzania and (v) New Zealand. Independent classification models were developed for each of these locations and where data permitted separate models within each location for natural and infrared illumination. Details of the hardware, datasets, model configuration and evaluation results are provided in order to provide a general assessment of *ClassifyMe* performance across diverse settings.

**S3.1: New England New South Wales, Australia**

**Overview:**

The New England region of New South Wales (NSW), Australia comprises extensive sheep stations in pastoral environments adjoining extensive sclerophyll bushland throughout the Great Dividing Range. The species cohort consists of a diverse mix of livestock, introduced and native animals. Camera trap monitoring in the region consists of either monitoring remnant vegetation on sheep stations or monitoring animal movements along vehicle access trails in state reserves and national parks. The dataset assessed represents the most frequent species or categories occurring at regional study sites for which it was possible to obtain annotations. More comprehensive sampling and species/category coverage is envisaged although the current capabilities provided by the *ClassifyMe* are already relevant and of significant assistance to research programs within this region.

| **Category** | **Natural Sample Size**  **(Training){Validation}[Test]** | **IR Sample Size**  **(Training){Validation}[Test]** |
| --- | --- | --- |
| Cat | (800){100}[100] | (800){100}[100] |
| Dog | (800){100}[100] | (800){100}[100] |
| Fox | (800){100}[100] | (800){100}[100] |
| Human | (800){100}[100] | (800){100}[100] |
| Macropod | (800){100}[100] | (800){100}[100] |
| Sheep | (800){100}[100] | (800){100}[100] |
| Vehicle | (800){100}[100] | (800){100}[100] |
| Other | (800){100}[100] | (800){100}[100] |
| NIL | (800){0}[100] | (800){0}[100] |

**Table A3.1.1: New England NSW, Australia dataset. Note the ‘Other’ dataset consisted of other animal categories of insufficient data to create a separate model category and included birds, cattle, echidnas, hares and rabbits. All images were captured using arrays of Scoutguard SG560KV or Reconyx HC600 Hyperfire cameras.**

**Model Training and Assessment Procedure:**

A model training dataset was formed by randomly sampling (via software) 800 images per image category in both the natural illumination and IR illumination classes. This process was done to ensure balanced numbers of samples per category in order to avoid model bias to a particular category. Separate models were developed for the natural and IR illumination classes. Data augmentation consisted of random horizontal flip, random vertical flip, random rotation of 5 degrees and random colour jitter. The YOLOv2 framework was used for model training, via the “AlexeyAB” Windows port: <https://github.com/AlexeyAB/darknet> . Select information is provided in Table S3.1.2 and the full YOLOv2 configuration file is provided as supplementary material (‘NewEnglandNat.cfg’) which is representative of the training procedure adopted across all models reported. Model assessment was performed by inspecting detection performance on the training dataset (Table S3.1.3), along with evaluation on the randomly held-out test datasets via confusion matrices (Tables S3.1.4 and Tables S3.1.5) and key model performance statistics (Table S3.1.6). Model assessment in the tables presented was based on *uniform* class probabilities of annotated test datasets specific to the environment. Balanced designs, with equal probability of each category allows estimation of performance metrics with no bias to a particular category.

**Model Training Information:**

|  | **Natural Illumination** | **IR illumination** |
| --- | --- | --- |
| **Batch Size:** | 64 | 64 |
| **Decay Rate:** | 0.0005 | 0.0005 |
| **Hours of Training:** | Approx. 31 hours | Approx. 36.5 hours |
| **Iterations (optimal weights):** | 22,700 | 26,600 |
| **Images Processed:** | 7,200 | 7,200 |
| **Learning Rate:** | 0.0001 | 0.0001 |
| **Momentum:** | 0.9 | 0.9 |

**Table S3.1.2: Model Training Information, New England NSW, Australia Model.**

| **Class** | **Natural Illumination**  **(Average Precision)** | **IR Illumination**  **(Average Precision)** |
| --- | --- | --- |
| Cat | 99.65% | 97.14% |
| Dog | 90.91% | 90.91% |
| Fox | 90.91% | 90.91% |
| Human | 90.91% | 90.91% |
| Macropod | 80.87% | 90.73% |
| Sheep | 86.46% | 79.93% |
| Vehicle | 100.00% | 90.91% |
| Other | 77.14% | 90.36% |

**Table S3.1.3: Detection Summary results: New England NSW model.**

| **Predicted** | **Actual** | | | | | | | | | | |
| --- | --- | --- | --- | --- | --- | --- | --- | --- | --- | --- | --- |
|  |  | Cat | Dog | Fox | Human | Macropod | NIL | Other | Sheep | Vehicle | *Precision* |
|  | Cat | 100 | 0 | 0 | 0 | 0 | 0 | 0 | 0 | 0 | 1.00 |
|  | Dog | 0 | 100 | 0 | 0 | 0 | 0 | 0 | 0 | 0 | 1.00 |
|  | Fox | 0 | 0 | 99 | 0 | 0 | 0 | 3 | 0 | 0 | 0.97 |
|  | Human | 0 | 0 | 0 | 100 | 0 | 0 | 0 | 0 | 0 | 1.00 |
|  | Macropod | 0 | 0 | 1 | 0 | 97 | 0 | 1 | 0 | 0 | 0.98 |
|  | NIL | 0 | 0 | 0 | 0 | 2 | 100 | 8 | 0 | 0 | 0.91 |
|  | Other | 0 | 0 | 0 | 0 | 0 | 0 | 91 | 0 | 0 | 1.00 |
|  | Sheep | 0 | 0 | 0 | 0 | 1 | 0 | 0 | 100 | 0 | 0.99 |
|  | Vehicle | 0 | 0 | 0 | 0 | 0 | 0 | 0 | 0 | 100 | 1.00 |
|  | *Recall* | 1.00 | 1.00 | 0.99 | 1.00 | 0.97 | 1.00 | 0.91 | 1.00 | 1.00 | **Overall**  **Model**  **Accuracy:**  0.99 |

**Table S3.1.4: Confusion Matrix: New England NSW model (natural illumination).**

| **Predicted** | **Actual** | | | | | | | | | | |
| --- | --- | --- | --- | --- | --- | --- | --- | --- | --- | --- | --- |
|  |  | Cat | Dog | Fox | Human | Macropod | NIL | Other | Sheep | Vehicle | *Precision* |
|  | Cat | 100 | 0 | 0 | 0 | 0 | 0 | 0 | 0 | 0 | 1.00 |
|  | Dog | 0 | 100 | 0 | 0 | 0 | 0 | 0 | 0 | 0 | 1.00 |
|  | Fox | 0 | 0 | 100 | 0 | 0 | 0 | 2 | 0 | 0 | 0.98 |
|  | Human | 0 | 0 | 0 | 100 | 0 | 0 | 0 | 0 | 0 | 1.00 |
|  | Macropod | 0 | 0 | 0 | 0 | 97 | 0 | 0 | 1 | 0 | 0.99 |
|  | NIL | 0 | 0 | 0 | 0 | 1 | 100 | 6 | 0 | 0 | 0.93 |
|  | Other | 0 | 0 | 0 | 0 | 2 | 0 | 92 | 0 | 0 | 0.98 |
|  | Sheep | 0 | 0 | 0 | 0 | 0 | 0 | 0 | 99 | 0 | 1.00 |
|  | Vehicle | 0 | 0 | 0 | 0 | 0 | 0 | 0 | 0 | 100 | 1.00 |
|  | *Recall* | 1.00 | 1.00 | 1.00 | 1.00 | 0.97 | 1.00 | 0.92 | 0.99 | 1.00 | **Overall**  **Model**  **Accuracy:**  0.99 |

**Table S3.1.5: Confusion Matrix: New England NSW model (IR illumination).**

| **Metric** | **Natural Illumination** | **IR Illumination** |
| --- | --- | --- |
| Overall Accuracy | 0.98556 | 0.98667 |
| Overall Accuracy Standard Error | 0.00398 | 0.00382 |
| 95% Confidence Interval | [0.97776,0.99335] | [0.97917,0.99416] |
| Error Rate | 0.01444 | 0.01333 |
| Matthews Correlation Coefficient | 0.98388 | 0.98506 |
| True Positive Rate (Macro) | 0.98556 | 0.98667 |
| True Positive Rate (Micro) | 0.98556 | 0.98667 |
| Positive Predictive Value (Macro) | 0.98655 | 0.98705 |
| Positive Predictive Value (Micro) | 0.98556 | 0.98667 |
| AUNP | 0.99187 | 0.9925 |

**Table S3.1.6: Test Statistics: New England NSW.**

**S3.2: Wisconsin, USA**

**Overview:**

Wisconsin is a US State located in the MidWest and Great Lakes Region. The state features a diverse range of geographical features and environments including mixed hardwood and coniferous forests. Data was sourced from the Snapshot Wisconsin project (<https://dnr.wi.gov/topic/research/projects/snapshot/>) which involves a state-wide camera-trap monitoring program utilising community volunteers to annotate image data using Zooniverse (<https://www.zooniverse.org>) with a data subset suitable for machine learning research made available by the University of Minnesota Digital Repository (<https://conservancy.umn.edu/handle/11299/199819>) [42]. Species and categories selected for inclusion in the *ClassifyMe* Wisconsin model are based on the frequency of images in the dataset available and guided by the data standard for development of public models as per Supplementary Material S2. The dataset was published under the Unversity of Minnesota Data Repository *Policies and Terms of Use* (<https://conservancy.umn.edu/pages/drum/policies/#terms-of-use> ).

| **Category** | **Natural Sample Size**  **(Training)[Test]** | **IR Sample Size**  **(Training)[Test]** |
| --- | --- | --- |
| Coyote | (1107) [100] | (1107) [100] |
| Deer | (1107) [100] | (1107) [100] |
| Elk | (1107) [100] | (1107) [100] |
| Hare | N/A | (1107) [100] |
| Raccoon | N/A | (1107) [100] |
| Squirrel | (1107) [100] | (1107) [100] |
| Turkey | (1107) [100] | N/A |
| Other | (1107) [100] | (1107) [100] |
| NIL | (1107)[100] | (1107)[100] |

**Table S3.2.1: Wisconsin, USA dataset. Note the ‘Other’ dataset consisted of other animal categories of insufficient data to create a separate model category and included porcupine, mink, fisher, bird, bobcat, cat, otter, small mammals, opossum and sandhill crane.**

**Model Training and Assessment Procedure:**

Model training and assessment was similar to that performed in Section S3.1 for the New England, NSW model. The key differences were the source dataset, the category coverage and the number of training images per category. Tables S.3.2.1 and S.3.2.2 provide key details on the model training performance and species coverage. The ‘Other’ category incorporated random samples from the porcupine, mink, fisher, bird, bobcat, cat, otter, small mammals, opossum and sandhill crane categories. Model assessment results are reported in Tables S3.2.3, S3.2.4, S3.2.5 and S3.2.6.

**Model Training Information:**

|  | **Natural Illumination** | **IR illumination** |
| --- | --- | --- |
| **Batch Size:** | 64 | 64 |
| **Decay Rate:** | 0.0005 | 0.0005 |
| **Hours of Training:** | Approx. 24.8 hours | Approx. 31.3 hours |
| **Iterations (optimal weights):** | 18,000 | 22,700 |
| **Images Processed:** | 6,642 | 7,749 |
| **Learning Rate:** | 0.0001 | 0.0001 |
| **Momentum:** | 0.9 | 0.9 |

**Table S3.2.2: Model Training Information, Wisconsin, USA Model.**

| **Class** | **Natural Illumination**  **(Average Precision)** | **IR Illumination**  **(Average Precision)** |
| --- | --- | --- |
| Coyote | 90.80% | 90.87% |
| Deer | 90.82% | 90.77% |
| Elk | 90.89% | 90.71% |
| Hare | N/A | 90.84% |
| Raccoon | N/A | 90.87% |
| Squirrel | 90.01% | 76.72% |
| Turkey | 89.83% | N/A |
| Other | 87.97% | 90.78% |

**Table S3.2.3: Detection Summary results: Wisconsin, USA model.**

| **Predicted** | **Actual** | | | | | | | | |
| --- | --- | --- | --- | --- | --- | --- | --- | --- | --- |
|  |  | Coyote | Deer | Elk | NIL | Other | Squirrel | Turkey | *Precision* |
|  | Coyote | 93 | 0 | 0 | 0 | 3 | 0 | 0 | 0.97 |
|  | Deer | 5 | 100 | 0 | 2 | 0 | 0 | 0 | 0.93 |
|  | Elk | 0 | 0 | 100 | 0 | 4 | 0 | 0 | 0.96 |
|  | NIL | 0 | 0 | 0 | 95 | 1 | 5 | 0 | 0.94 |
|  | Other | 1 | 0 | 0 | 1 | 91 | 1 | 0 | 0.97 |
|  | Squirrel | 0 | 0 | 0 | 1 | 0 | 92 | 0 | 0.99 |
|  | Turkey | 1 | 0 | 0 | 1 | 1 | 2 | 100 | 0.95 |
|  | *Recall* | 0.93 | 1.00 | 0.86 | 0.95 | 0.91 | 0.92 | 1.00 | **Overall**  **Model**  **Accuracy:**  0.96 |

**Table S3.2.4: Confusion Matrix: Wisconsin, USA model (natural illumination).**

| **Predicted** | **Actual** | | | | | | | | | |
| --- | --- | --- | --- | --- | --- | --- | --- | --- | --- | --- |
|  |  | Coyote | Deer | Elk | Hare | NIL | Other | Racoon | Squirrel | *Precision* |
|  | Coyote | 94 | 0 | 0 | 0 | 1 | 0 | 0 | 0 | 0.99 |
|  | Deer | 2 | 98 | 0 | 0 | 0 | 0 | 0 | 0 | 0.98 |
|  | Elk | 0 | 0 | 100 | 0 | 0 | 0 | 0 | 0 | 1.00 |
|  | Hare | 0 | 1 | 0 | 100 | 1 | 0 | 0 | 0 | 0.98 |
|  | NIL | 0 | 1 | 0 | 0 | 95 | 0 | 0 | 1 | 0.98 |
|  | Other | 3 | 0 | 0 | 0 | 2 | 98 | 0 | 0 | 1.00 |
|  | Raccoon | 1 | 0 | 0 | 0 | 1 | 1 | 100 | 0 | 0.97 |
|  | Squirrel | 0 | 0 | 0 | 0 | 0 | 1 | 0 | 99 | 0.99 |
|  | *Recall* | 0.94 | 0.98 | 1.00 | 1.00 | 0.95 | 0.98 | 1.00 | 0.99 | **Overall**  **Model**  **Accuracy:**  0.98 |

**Table S3.2.5: Confusion Matrix: Wisconsin, USA model (IR illumination).**

| **Metric** | **Natural Illumination** | **IR Illumination** |
| --- | --- | --- |
| Overall Accuracy | 0.95857 | 0.98 |
| Overall Accuracy Standard Error | 0.00753 | 0.00495 |
| 95% Confidence Interval | [0.94381,0.97333] | [0.9703,0.9897] |
| Error Rate | 0.04143 | 0.02 |
| Matthews Correlation Coefficient | 0.95188 | 0.97719 |
| True Positive Rate (Macro) | 0.95857 | 0.98 |
| True Positive Rate (Micro) | 0.95857 | 0.98 |
| Positive Predictive Value (Macro) | 0.95931 | 0.9802 |
| Positive Predictive Value (Micro) | 0.95857 | 0.98 |
| AUNP | 0.97583 | 0.98857 |

**Table S3.2.6: Test Statistics: Wisconsin, USA.**

**S3.3: South Western, USA**

**Overview:**

The South Western, USA comprises camera trapping data from the Caltech Camera Traps dataset (<https://beerys.github.io/CaltechCameraTraps/>) with the specific data used for building the model provided by the Labeled Information Library of Alexandria: Biology and Conservation (<http://lila.science/datasets/caltech-camera-traps>) and has been previously reported [43]. The full data set contains 243,187 images from 140 camera locations thereby representing a diverse range of environments and conditions. A random subset of the data was selected to match the *ClassifyMe* data requirements as close as possible as per Supplementary Material S2. The Caltech camera trap dataset was published under the following Community Data Licence Agreement (<https://cdla.io/permissive-1-0/> ) .

| **Category** | **Natural Sample Size**  **(Training)[Test]** | **IR Sample Size**  **(Training)[Test]** |
| --- | --- | --- |
| Bobcat | (1107) [100] | (1107) [100] |
| Cat | (1107) [100] | (1107) [100] |
| Coyote | (1107) [100] | (1107) [100] |
| Dog | (1107)[100] | N/A |
| Opossum | N/A | (1107) [100] |
| Other | (1107) [100] | (1107) [100] |
| Rabbit | (1107)[100] | (1107)[100] |
| Raccoon | N/A | (1107) [100] |
| Squirrel | (1107)[100] | N/A |
| NIL | (0)[100] | (0)[100] |

**Table S3.3.1: South Western, USA dataset. Note the ‘Other’ dataset consisted of other animal categories of insufficient data to create a separate model category and included skunk, deer, raccoon, opossum, car. The ‘NIL’ category was unavailable for the natural light dataset and not used in either model training or testing.**

**Model Training Information:**

|  | **Natural Illumination** | **IR illumination** |
| --- | --- | --- |
| **Batch Size:** | 64 | 64 |
| **Decay Rate:** | 0.0005 | 0.0005 |
| **Hours of Training:** | Approx. 26 hours | Approx. 26 hours |
| **Iterations (optimal weights):** | 18,000 | 18,100 |
| **Images Processed:** | 6,642 | 7,749 |
| **Learning Rate:** | 0.0001 | 0.0001 |
| **Momentum:** | 0.9 | 0.9 |

**Table S3.3.2: Model Training Information, South Western, USA Model.**

| **Class** | **Natural Illumination**  **(Average Precision)** | **IR Illumination**  **(Average Precision)** |
| --- | --- | --- |
| Bobcat | 90.66% | 90.75% |
| Cat | 90.81% | 90.73% |
| Coyote | 90.90% | 90.82% |
| Dog | 90.88% | N/A |
| Opossum | N/A | 90.84% |
| Other | 78.02% | 90.41% |
| Rabbit | 90.08% | 90.81% |
| Raccoon | N/A | 89.18% |
| Squirrel | 89.93% | N/A |

**Table S3.3.3: Detection Summary results: South Western, USA model.**

| **Predicted** | **Actual** | | | | | | | | | |
| --- | --- | --- | --- | --- | --- | --- | --- | --- | --- | --- |
|  |  | Bobcat | Cat | Coyote | Dog | NIL | Other | Rabbit | Squirrel | *Precision* |
|  | Bobcat | 97 | 0 | 0 | 0 | 0 | 0 | 1 | 0 | 0.99 |
|  | Cat | 1 | 99 | 0 | 1 | 0 | 0 | 0 | 0 | 0.98 |
|  | Coyote | 1 | 0 | 99 | 0 | 1 | 0 | 0 | 0 | 0.99 |
|  | Dog | 0 | 0 | 0 | 99 | 0 | 0 | 0 | 0 | 1.00 |
|  | NIL | 0 | 0 | 0 | 0 | 96 | 0 | 0 | 0 | 1.00 |
|  | Other | 0 | 0 | 0 | 0 | 1 | 97 | 0 | 0 | 1.00 |
|  | Rabbit | 0 | 0 | 0 | 0 | 0 | 0 | 94 | 2 | 0.98 |
|  | Squirrel | 0 | 0 | 0 | 0 | 2 | 0 | 1 | 93 | 0.99 |
|  | *Recall* | 0.97 | 1.00 | 0.99 | 0.99 | 0.96 | 0.97 | 0.94 | 0.93 | **Overall**  **Model**  **Accuracy:**  0.97 |

**Table S3.3.4: Confusion Matrix: South Western, USA model (natural illumination)**

| **Predicted** | **Actual** | | | | | | | | | |
| --- | --- | --- | --- | --- | --- | --- | --- | --- | --- | --- |
|  |  | Bobcat | Cat | Coyote | Opossum | Other | NIL | Rabbit | Raccoon | *Precision* |
|  | Bobcat | 99 | 1 | 0 | 0 | 0 | 0 | 0 | 1 | 0.99 |
|  | Cat | 0 | 98 | 1 | 0 | 0 | 0 | 0 | 0 | 0.99 |
|  | Coyote | 0 | 0 | 98 | 0 | 0 | 0 | 0 | 0 | 1.00 |
|  | Opossum | 0 | 0 | 0 | 100 | 0 | 0 | 0 | 0 | 1.00 |
|  | Other | 0 | 0 | 1 | 0 | 96 | 2 | 0 | 0 | 0.97 |
|  | NIL | 0 | 1 | 0 | 0 | 0 | 98 | 0 | 0 | 0.99 |
|  | Rabbit | 1 | 0 | 0 | 0 | 2 | 0 | 100 | 0 | 0.97 |
|  | Raccoon | 0 | 0 | 0 | 0 | 0 | 0 | 0 | 99 | 1.00 |
|  | *Recall* | 0.99 | 0.98 | 0.98 | 1.00 | 0.96 | 0.98 | 1.00 | 0.99 | **Overall**  **Model**  **Accuracy:**  0.99 |

**Table S3.3.5: Confusion Matrix: South Western, USA model (IR illumination).**

| **Metric** | **Natural Illumination** | **IR Illumination** |
| --- | --- | --- |
| Overall Accuracy | 0.9675 | 0.985 |
| Overall Accuracy Standard Error | 0.00627 | 0.0043 |
| 95% Confidence Interval | [0.95521,0.97979] | [0.97658,0.99342] |
| Error Rate | 0.0325 | 0.015 |
| Matthews Correlation Coefficient | 0.96298 | 0.98287 |
| True Positive Rate (Macro) | 0.9675 | 0.985 |
| True Positive Rate (Micro) | 0.9675 | 0.985 |
| Positive Predictive Value (Macro) | 0.96883 | 0.98512 |
| Positive Predictive Value (Micro) | 0.9675 | 0.985 |
| AUNP | 0.98143 | 0.99143 |

**Table S3.3.6: Test Statistics: South Western, USA.**

**S3.4: Serengeti Plains, Tanzania**

**Overview:**

The Snapshot Serengeti project is a well-known project featuring approximately 3.2 million images and annotated by volunteers using Zooniverse (<https://www.zooniverse.org>). The dataset features camera trap images of 40 mammal species on the African savanna [23]. We utilised the dataset provided by the Labeled Information Library of Alexandria: Biology and Conservation (<http://lila.science/datasets/snapshot-serengeti>) in order to access annotated data suitable machine learning object detection. The Snapshot Serengeti dataset is highly unbalanced across categories which is reflective of the data recorded by the camera traps in operational environments. Categories selected for inclusion in the *ClassifyMe* Serengeti model were based on the most frequent categories and also meeting the minimum number of annotated images to ensure model quality control. Camera trap images included both natural and flash illumination, the *ClassifyMe* Serengeti model does not presently distinguish between these two illumination categories. Future work on the model will focus on the expansion of species and categories covered and potentially illumination specific models. This dataset was accessed under the Community Data Licence Agreement (<https://cdla.io/permissive-1-0/>).

| **Category** | **Sample Size**  **(Training){Validation}[Test]** |
| --- | --- |
| Baboon | (1400){175}[175] |
| Cheetah | (1400){175}[175] |
| Elephant | (1400){175}[175] |
| Gazelle | (1400){175}[175] |
| Giraffe | (1400){175}[175] |
| Human | (1400) {175}[175] |
| Hyena | (1400){175}[175] |
| Lion | (1400){175}[175] |
| Wildebeest | (1400){175}[175] |
| Zebra | (1400){175}[175] |
| NIL | (0){0}[175] |

**Table S3.4.1: Serengeti, Tanzania dataset.**

**Model Training Information:**

| **Batch Size:** | 64 |
| --- | --- |
| **Decay Rate:** | 0.0005 |
| **Hours of Training:** | Approx. 39.5 hours |
| **Iterations (optimal weights):** | 28,000 |
| **Images Processed:** | 14,000 |
| **Learning Rate:** | 0.0001 |
| **Momentum:** | 0.9 |

**Table S3.4.2: Model Training Information, Serengeti, Tanzania Model.**

| **Class** | **Average Precision** |
| --- | --- |
| Baboon | 77.43% |
| Cheetah | 88.76% |
| Elephant | 79.45% |
| Gazelle | 81.16% |
| Giraffe | 80.41% |
| Human | 90.08% |
| Hyena | 90.91% |
| Lion | 90.26% |
| Wildebeest | 79.62% |
| Zebra | 80.92% |

**Table S3.4.3: Detection Summary results: Serengeti, Tanzania model.**

| **Predicted** | **Actual** | | | | | | | | | | | | |
| --- | --- | --- | --- | --- | --- | --- | --- | --- | --- | --- | --- | --- | --- |
|  |  | Baboon | Cheetah | Elephant | Gazelle | Giraffe | Human | Hyena | Lion | NIL | WBeest | Zebra | *Precision* |
|  | Baboon | 174 | 0 | 0 | 0 | 1 | 1 | 1 | 0 | 1 | 0 | 0 | 0.98 |
|  | Cheetah | 0 | 174 | 0 | 0 | 0 | 0 | 0 | 1 | 0 | 0 | 0 | 0.99 |
|  | Elephant | 0 | 0 | 175 | 0 | 0 | 1 | 0 | 0 | 0 | 0 | 0 | 0.99 |
|  | Gazelle | 0 | 0 | 0 | 172 | 0 | 0 | 0 | 0 | 0 | 0 | 0 | 1.00 |
|  | Giraffe | 0 | 0 | 0 | 0 | 174 | 0 | 0 | 0 | 3 | 0 | 0 | 0.98 |
|  | Human | 0 | 0 | 0 | 1 | 0 | 170 | 0 | 0 | 0 | 0 | 0 | 0.99 |
|  | Hyena | 0 | 0 | 0 | 0 | 0 | 0 | 174 | 1 | 0 | 0 | 0 | 0.99 |
|  | Lion | 0 | 0 | 0 | 0 | 0 | 0 | 0 | 173 | 0 | 0 | 0 | 1.00 |
|  | NIL | 1 | 0 | 0 | 2 | 0 | 3 | 0 | 0 | 170 | 0 | 0 | 0.97 |
|  | WBeest | 0 | 0 | 0 | 0 | 0 | 0 | 0 | 0 | 0 | 175 | 0 | 1.00 |
|  | Zebra | 0 | 1 | 0 | 0 | 0 | 0 | 0 | 0 | 1 | 0 | 175 | 0.99 |
|  | *Recall* | 0.99 | 0.99 | 1.00 | 0.98 | 0.99 | 0.97 | 0.99 | 0.99 | 0.97 | 1.00 | 1.00 | **Overall**  **Model**  **Accuracy**  0.99 |

**Table S3.4.4: Confusion Matrix: Serengeti, Tanzania model. WBeest denotes *Wildebeest*.**

| **Metric** | **Magnitude** |
| --- | --- |
| Overall Accuracy | 0.99013 |
| Overall Accuracy Standard Error | 0.00225 |
| 95% Confidence Interval | [0.98571,0.99455] |
| Error Rate | 0.00987 |
| Matthews Correlation Coefficient | 0.98915 |
| True Positive Rate (Macro) | 0.99013 |
| True Positive Rate (Micro) | 0.99013 |
| Positive Predictive Value (Macro) | 0.99020 |
| Positive Predictive Value (Micro) | 0.99013 |
| AUNP | 0.99457 |

**Table S3.4.5: Test Statistics: Serengeti, Tanzania**

**S3.5: New Zealand**

**Overview:**

The New Zealand data set consists of camera trapping data from the *Kiwi Rescue* program [41]. The *Kiwi Rescue* program represents an inter-institutional collaboration of government and academic scientists focused on the recovery of Kiwi (*Apteryx* spp.) populations. Camera traps were utilised in this program for predator surveillance and monitoring of Kiwi populations. Image data was obtained from sites across New Zealand, particularly Fiordland in the South Island. The dataset was provided by A. Glen of Manaaki Whenua – Landcare Research, Auckland, New Zealand and consisted of 5,228 annotated camera trap images (9.57GB) relevant to New Zealand Kiwi population monitoring. Due to the limited number of images per category across illumination classes, the data set was combined into monochrome format by transforming RGB colour images to grey-scale images. Therefore, only a single model was developed and evaluated for the NZ dataset.

| **Category** | **Sample Size**  **(Training){Validation}[Test]** |
| --- | --- |
| Bird | (714){94}[20] |
| Cat | (174){21}[20] |
| Hedgehog | (765){101}[20] |
| Kiwi | (837){110}[20] |
| Sheep | (940){125}[20] |
| Stoat | (502) {66}[20] |
| NIL | (523){65}[20] |

**Table S3.5.1: New Zealand dataset.**

**Model Training Information:**

| **Batch Size:** | 64 |
| --- | --- |
| **Decay Rate:** | 0.0005 |
| **Hours of Training:** | Approx. 24.2 hours |
| **Iterations (optimal weights):** | 19,400 |
| **Images Processed:** | 4,455 |
| **Learning Rate:** | 0.0001 |
| **Momentum:** | 0.9 |

**Table S3.5.2: Model Training Information, New Zealand Model.**

| **Class** | **Average Precision** |
| --- | --- |
| Bird | 80.61% |
| Cat | 90.91% |
| Hedgehog | 90.91% |
| Kiwi | 90.91% |
| Sheep | 90.43% |
| Stoat | 90.91% |

**Table S3.5.3: Detection Summary results: New Zealand Model.**

| **Predicted** | **Actual** | | | | | | | | |
| --- | --- | --- | --- | --- | --- | --- | --- | --- | --- |
|  |  | Bird | Cat | Hedgehog | Kiwi | NIL | Sheep | Stoat | *Precision* |
|  | Bird | 17 | 0 | 0 | 0 | 0 | 0 | 0 | 1.00 |
|  | Cat | 0 | 20 | 0 | 0 | 0 | 0 | 0 | 1.00 |
|  | Hedgehog | 0 | 0 | 20 | 0 | 0 | 0 | 0 | 1.00 |
|  | Kiwi | 0 | 0 | 0 | 20 | 0 | 0 | 0 | 1.00 |
|  | NIL | 3 | 0 | 0 | 0 | 20 | 0 | 0 | 0.87 |
|  | Sheep | 0 | 0 | 0 | 0 | 0 | 20 | 0 | 1.00 |
|  | Stoat | 0 | 0 | 0 | 0 | 0 | 0 | 20 | 1.00 |
|  | *Recall* | 0.85 | 1.00 | 1.00 | 1.00 | 1.00 | 1.00 | 1.00 | **Overall**  **Model**  **Accuracy:**  0.98 |

**Table S3.5.4: Confusion Matrix: New Zealand Model.**

| **Metric** | **Magnitude** |
| --- | --- |
| Overall Accuracy | 0.97857 |
| Overall Accuracy Standard Error | 0.01224 |
| 95% Confidence Interval | [0.95458,1.00256] |
| Error Rate | 0.02143 |
| Matthews Correlation Coefficient | 0.97552 |
| True Positive Rate (Macro) | 0.97857 |
| True Positive Rate (Micro) | 0.97857 |
| Positive Predictive Value (Macro) | 0.98137 |
| Positive Predictive Value (Micro) | 0.97857 |
| AUNP | 0.9875 |

**Table S3.5.5: Test Statistics: New Zealand.**

**S3.6: Computational Performance Evaluations**

| **Processor** | **Mode RAM** | **Dataset** | **Number of Images (Testing)** | **Mode (GPU/CPU)** | **Total Time**  **(seconds)** | **Rate (Images/Second)** |
| --- | --- | --- | --- | --- | --- | --- |
| I7-8700 CPU @ 3.20 GHz | 16 GB | New England, NSW, Australia, Natural Illumination | 640 | GPU | 440 | 1.455 |
| I7-8700 CPU @ 3.20 GHz | 16 GB | New England, NSW, Australia, IR | 640 | GPU | 436 | 1.468 |
| I7-8700 CPU @ 3.20 GHz | 16 GB | Wisconsin, USA, Natural Illumination | 738 | GPU | 513 | 1.489 |
| I7-8700 CPU @ 3.20 GHz | 16 GB | Wisconsin, USA, IR | 861 | GPU | 644 | 1.337 |
| I7-8700 CPU @ 3.20 GHz | 16 GB | South Western, USA, Natural Illumination | 861 | GPU | 613 | 1.405 |
| I7-8700 CPU @ 3.20 GHz | 16 GB | South Western, USA, IR | 861 | GPU | 779 | 1.105 |
| I7-8700 CPU @ 3.20 GHz | 16 GB | Serengeti, Tanzania | 2500 | GPU | 1784 | 1.401 |
| I7-8700 CPU @ 3.20 GHz | 16 GB | New Zealand, Monochrome | 120 | GPU | 92 | 1.304 |

**Table S3.6.1: ClassifyMe computational performance evaluation metrics.**
