# Supplementary material for: ClassifyMe: A Field-Scouting Software for the Identification of Wildlife in Camera Trap Images
Source: Animals (Basel). 2019 Dec 27;10(1):58. doi: 10.3390/ani10010058 (PMC7022311; doi:10.3390/ani10010058)
Supplement: Supplementary file 1 [file animals-10-00058-s001.zip › sup/ClassifyMe_SupplementaryMaterial S2.docx]

**Supplementary Material S2: Data Presentation Standard for *ClassifyMe* Software**

In order to develop *ClassifyMe* models for each site, our team needs the following information. Please note this form provides instructions on how we would like data to be presented for addition to the model. This document is not confirmation that UNE or NSW DPI will undertake or commit to development of a specific model. Provision of data to UNE and DPI is gratefully accepted and will not be used or provided to any other parties. Data will only be used for model development and refinement. The number of images for each category is our preferred minimum, lower numbers of images or unequal numbers of images per category or illumination class doesn’t necessarily prevent us developing a model We will assess the data provided and advise you if we cannot provide a model for your data.

**Step 1. Model Name**

Please provide a suggested name for the model. This should be reflective of the location where the camera trap data was collected. Please try to limit the name to 20 characters or less e.g. CoffsCoast.

**Step 2. Metadata**

Please provide the following details:

1. Principle Scientist/Senior Manager approving the provision of data
2. Institution or agency with ownership rights to data
3. Site of data collection
4. Date of collection
5. Camera trap models
6. Details of settings and placement of camera traps
7. List of the species of interest

**Step 3. Naming Convention**

There is no specific naming convention, but please don’t send images with just the filename used by the camera trap. We suggest renaming files using RENAMER to a simple code that you will recognise and so that each image has a unique identifier name.

**Step 4. Model Data**

We require a dictionary (folders) of all key species/category. In each folder, we require sub- folders for each illumination class e.g. Day (natural illumination), Night (IR illumination), and if used Night (*white-flash illumination). Please only organise to Genus level for species/categories. We also require false positive images which just show the landscape with no animals/objects in the frame.

These sub-folders should be further partitioned into folders describing each species/category, for example;

1. Coffs Coast/Night Infra Red/Macropod – 1230 photos
2. Coffs Coast/Day/Macropod – 1230 photos
3. Coffs Coast/ Day/False Positive – 1230 photos
4. Coffs Coast/ Night/False Positive – 1230 photos

We require equal numbers of images for each dataset i.e. each species/category per illumination class needs to have 1230 images, if you have more that is helpful but please keep the number of images consistent for each category.

**Step 5: Send Data, Forms and Information**

Please contact Paul Meek to organise model development in order for us to organise our data science team resources. Data will need to be uploaded to a cloud service or sent via thumb drive or hard drive.

**Additional Notes**

We currently focus on processing camera trap images where the camera has been set in the horizontal alignment and as such the camera trap PIR is horizontal with the horizon.

Please do not reduce resolution of the original camera trap images or perform any other form of image processing. Consult with us first if you have special processing requirements.

Random sampling across a variety of contexts, image backgrounds, poses, distances etc. is preferred if possible to make the system more robust.

For species/categories with insufficient data, the category ‘Other’ can be provided. This will ensure *ClassifyMe* is trained to detect animals/birds/interesting object categories in general.

Please do not send any images of humans for privacy reasons.

If you have individually recognisable animals and have enough images of them in natural and infra-red illumination, we would ask that you provide these animals in separate folder categories for example Kakudu /AnimalName/Day/Dingo and Kakudu/AnimalName/Night/Dingo. We need as many images per individual as possible but not exceeding 1000. These same animals can be included in the main data set as well.

* If you have IR and White flash photos for the night photos we need separation of IR and white flash and again 1230 images of each sub-category.

Please contact:

Dr Paul Meek
paul.meek@dpi.nsw.gov.au
